# Recombination in Eukaryotic Single Stranded DNA Viruses

**DOI:** 10.3390/v3091699

**Published:** 2011-09-13

**Authors:** Darren P. Martin, Philippe Biagini, Pierre Lefeuvre, Michael Golden, Philippe Roumagnac, Arvind Varsani

**Affiliations:** 1 Computational Biology Group, Institute of Infectious Diseases and Molecular Medicine, University of Cape Town, Cape Town 4579, South Africa; E-Mail: GLDMIC011@uct.ac.za; 2 UMR CNRS 6578 Anthropologie Bioculturelle, Equipe “Emergence et co-évolution virale”, Etablissement Français du Sang Alpes-Méditerranée, Université de la Méditerranée, 27 Bd. Jean Moulin, 13005 Marseille, France; E-Mail: philippe.biagini@univmed.fr; 3 CIRAD, UMR 53 PVBMT CIRAD-Université de la Réunion, Pôle de Protection des Plantes, Ligne Paradis, 97410, Saint Pierre, La Réunion, France; E-Mail: pierre.lefeuvre@gmail.com; 4 CIRAD, UMR BGPI, TA A-54/K, Campus International de Montferrier-Baillarguet, 34398 Montpellier, France; E-Mail: philippe.roumagnac@cirad.fr; 5 Electron Microscope Unit, University of Cape Town, Rondebosch, Cape Town 7701, South Africa; E-Mail: arvind.varsani@canterbury.ac.nz; 6 Biomolecular Interaction Centre, University of Canterbury, Private Bag 4800, Christchurch 8140, New Zealand; 7 School of Biological Sciences, University of Canterbury, Private Bag 4800, Christchurch 8140, New Zealand

**Keywords:** parvovirus, geminivirus, anellovirus, circovirus, nanovirus

## Abstract

Although single stranded (ss) DNA viruses that infect humans and their domesticated animals do not generally cause major diseases, the arthropod borne ssDNA viruses of plants do, and as a result seriously constrain food production in most temperate regions of the world. Besides the well known plant and animal-infecting ssDNA viruses, it has recently become apparent through metagenomic surveys of ssDNA molecules that there also exist large numbers of other diverse ssDNA viruses within almost all terrestrial and aquatic environments. The host ranges of these viruses probably span the tree of life and they are likely to be important components of global ecosystems. Various lines of evidence suggest that a pivotal evolutionary process during the generation of this global ssDNA virus diversity has probably been genetic recombination. High rates of homologous recombination, non-homologous recombination and genome component reassortment are known to occur within and between various different ssDNA virus species and we look here at the various roles that these different types of recombination may play, both in the day-to-day biology, and in the longer term evolution, of these viruses. We specifically focus on the ecological, biochemical and selective factors underlying patterns of genetic exchange detectable amongst the ssDNA viruses and discuss how these should all be considered when assessing the adaptive value of recombination during ssDNA virus evolution.

## 1. Introduction

**Figure 1.**
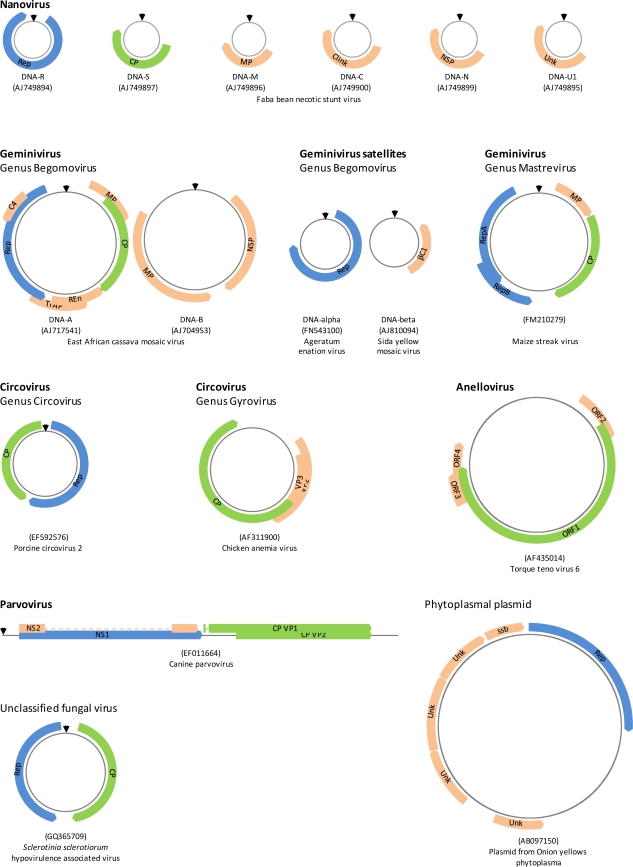
Single stranded (ss) DNA virus genomes and related replicons. Many ssDNA viruses have homologous replication associated protein genes (Rep, indicated in blue) that are crucial for the initiation of either rolling circle (for circular molecules) or rolling hairpin (for linear molecules) replication. Wherever replication origins are known they are indicated by black arrows. CP = coat protein (although not all obviously homologous they are all represented in green). MP = movement protein; Clink = Cell cycle regulatory protein; NSP = nuclear shuttle protein; Unk = unknown function; Trap = Transcription activator protein; Ren = Replication enhancer protein, ssb = single stranded DNA binding protein; VP = virion/viral protein; NS = non-structural protein.

Single stranded (ss) DNA viruses infect animals, plants, fungi and bacteria and are known to cause a variety of diseases in domesticated plants and animals. Their genomes can be linear or circular, multi- or single component and are all smaller than 9 Kb in length (Figure 1). There are presently only seven recognized ssDNA virus families including three that infect animals (the *Anelloviridae*, *Circoviridae* and *Parvoviridae*), two that infect plants, (the *Geminiviridae* and *Nanoviridae*), and two that infect prokaryotes (the *Microviridae* and *Inoviridae*). Falling outside these groups are an enormous variety of what appear to be either ssDNA viruses or plasmids that have been recently discovered during metagenomic surveys of terrestrial and aquatic environments [1–7]. While many of these diverse replicons are clearly related to known ssDNA viruses, they are so divergent that they likely represent tens if not hundreds of entirely new ssDNA virus families. Although it is unknown what species most of these replicons might infect, their sheer number and diversity suggests that their hosts may collectively span the entire tree of life.

**Figure 2.**
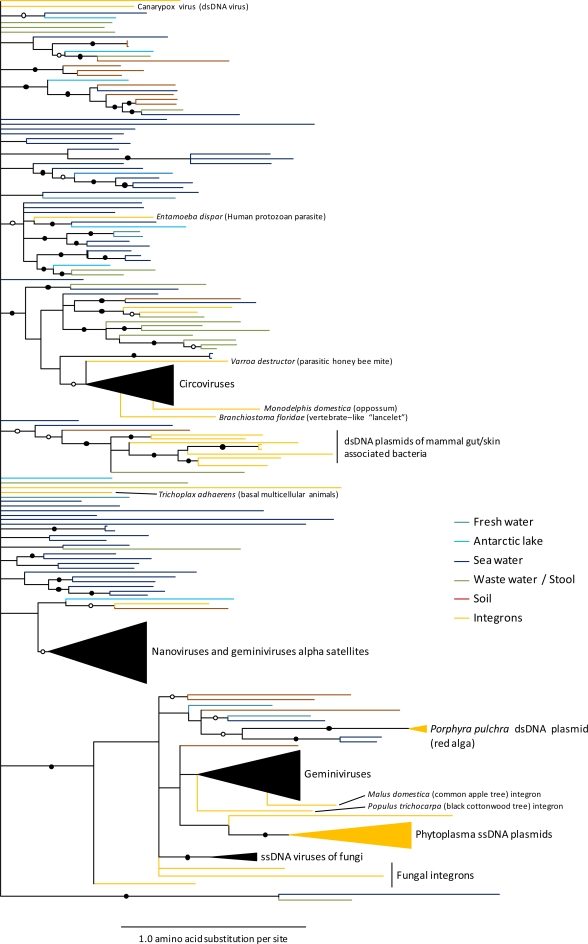
Phylogenetic tree depicting the evolutionary relationships between the replication associated proteins of various rolling circle replicons. Included here are sequences from known viruses (such as the geminiviruses, nanoviruses and circoviruses; black triangles representing large groups of closely related sequences), plasmids (orange branches/triangle), sequences found integrated within eukaryote genomes (orange branches with species names given), and potential virus genome sequences discovered during metagenomic screens of aqueous environments (blue branches) and soil (brown branches). An initial tree was constructed using a maximum likelihood approach with the JTT amino acid substitution model. This initial tree was then subdivided into four subtrees and the sequences represented in each of these subtrees were used in BAli-Phy (a MCMC method for simultaneous Bayesian estimation of alignment and phylogeny; [26]) to infer new subtrees taking alignment uncertainty into account. Branches indicated by a filled black circle represent a posterior support probability >0.95, whereas open circles represent a posterior support probability of >0.80. Branches with a posterior support probability <0.50 have been collapsed.

At least some of the major ssDNA virus groups are evolutionarily related [4]. The most obvious evolutionary links can be found amongst the circular eukaryote infecting ssDNA viruses that express a conserved replication associated protein (called Rep) and replicate via a rolling circle mechanism. The genes encoding this protein and the virion strand origin of replication that it interacts with to initiate rolling circle replication (RCR), seem to be quite highly conserved across viruses in the families *Geminiviridae*, *Circoviridae* and *Nanoviridae*, two families of bacterial plasmids and many of the unclassified circular ssDNA molecules directly sequenced from the environment (Figure 2; [1–5,7,8]).

Although reasonably tenuous, some evidence exists that the Rep proteins of the geminiviruses, nanoviruses and circoviruses also share distant evolutionary relationships with the NS1 proteins of linear animal infecting ssDNA viruses in the family *Parvoviridae*, the Rep proteins of circular ssDNA prokaryote infecting viruses in the family *Microviridae* [9] and various eubacterial and archeabacterial ssDNA plasmid families. For example, although geminivirus and microvirus Rep proteins display no obvious sequence similarity, they both play very similar roles during RCR and structurally contain strikingly similar arrangements of key RCR associated motifs [9–12].

Despite the possibility that the evolutionary history of the ssDNA viruses may span that of life on earth [13,14], the members of all the established ssDNA virus families are apparently capable of extremely rapid evolution. Besides displaying nucleotide substitution rates between 10^−4^ and 10^−3^ substitutions per site per year—rates closer to those of RNA viruses than double stranded DNA viruses [5,15–24]—it is also evident that frequent recombination has featured prominently in the evolution of the ssDNA viruses.

It has in fact been speculated that both the circovirus [25] and geminivirus [4] families may have originated through recombination. While it has been proposed that the geminiviruses first arose when a recombination event married the coat protein gene of an icosahedral plant ssRNA virus with the Rep gene of a ssDNA bacterial plasmid (such as those associated with phytoplasmas [4]), it has also been proposed that the circoviruses may have arisen through recombination between a nanovirus and a ssRNA picorna-like virus [25].

While not entirely implausible, neither of these recombinant origin hypotheses are strongly supported by the available nucleotide sequence data. Whereas the geminivirus coat protein has a fold that is superficially similar to those of some ssRNA icosahedral viruses, it displays absolutely no detectable sequence similarity to any of these coat proteins. Also, while the geminivirus Rep is quite closely related to that of a group of phytoplasmal ssDNA plasmids, it is just as closely related to Reps expressed by numerous environmental ssDNA molecules (Figure 2). In fact, the phylogenetic relationships of various phytoplasmal plasmid genes indicate that a far more parsimonious explanation for the relationship between geminivirus and phytoplasmal Rep genes is that an ancestral phytoplasmal plasmid obtained its Rep gene through recombination with an ancient geminivirus-like ssDNA virus [27].

The exact sources of the sequences that make up the circoviral Rep are similarly obscure. Whereas the N-terminus region of the circovirus Rep is clearly homologous to the N-terminal region of nanovirus and geminivirus Reps, the C terminal half of the gene appears to be homologous to a fragment of the 2C protein of picorna-like viruses [25]. However, the C-terminus regions of numerous Rep sequences encoded by environmental ssDNAs also have detectable degrees of similarity to picorna-like virus 2C proteins. This indicates that the apparent inter-familial recombination event detected within the circovirus genomes [25] must have either occurred prior to the emergence of the first circovirus, or the picorna-like virus sequences that differentiate the circovirus Rep sequences from those of nanoviruses and geminiviruses must have been distributed by secondary recombination events amongst divergent groups of ssDNA replicons [28].

Far better supported by the available sequence data is extensive evidence of recombination events which have resulted in the generation of new genera, species and strains of geminiviruses [29–36], circoviruses [37–45], nanoviruses [40,46,47], anelloviruses [48–50] and parvoviruses [40,51–55]. The types of recombination that occur within these families include homologous recombination during which sequences within one genome are replaced with homologous sequences from another genome, non-homologous recombination during which genome regions get rearranged, duplicated, deleted or are inserted into the genomes of host cells, and reassortment (or pseudo recombination) during which whole genome components of multi-component ssDNA virus genomes get exchanged between strains or species.

## 2. Homologous Recombination between ssDNA Virus Genomes

Mechanisms of homologous recombination in ssDNA viruses are still quite poorly characterized but are most probably strongly influenced by the ways in which these viruses replicate. Amongst the eukaryote infecting ssDNA viruses, genomic replication and recombination processes have been the most thoroughly analyzed in the parvoviruses, geminiviruses and circoviruses. This is because these viruses generally replicate to very high titers and numerous techniques are available for easily initiating infections from cloned virus genomes. While it is very difficult to reconstitute infections from multiple cloned nanovirus genome components [56], in the anelloviruses despite *in vitro* infections being achievable with either virus particles [57] or viral genome clones [58,59] no suitable cell culture systems are available in which cloned viruses will replicate to high enough titers to study the mechanistic details of their replication and recombination.

### 2.1. Replication of ssDNA Viruses

Whereas all of the circular eukaryotic ssDNA replicons that express Rep homologues are likely to replicate via RCR ([60–64] reviewed in Gutierrez *et al.* [65]), the linear genomes of parvoviruses are generally replicated by variants of a so-called “rolling hairpin replication” (RHR) mechanism [66–68].

**Figure 3.**
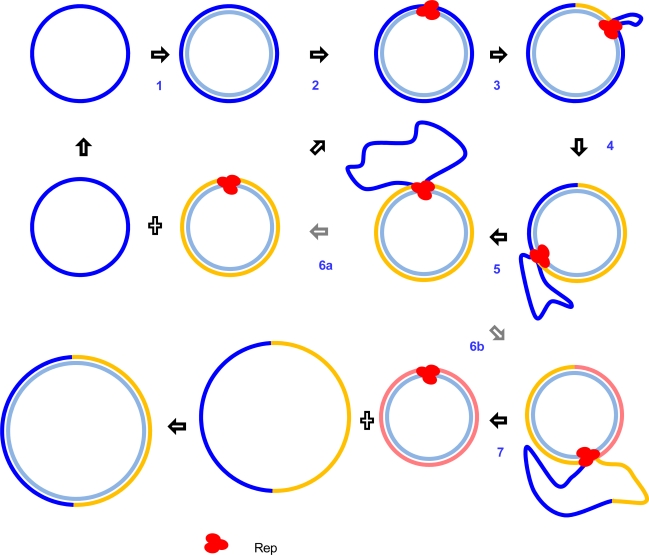
Rolling-circle replication in the geminivirus, Abutilon mosaic virus. The circular unencapsidated parental virion strand (in dark blue) is converted to double stranded DNA by host DNA polymerases (a process that is also primed by a host derived primer molecule; Step 1). Rep (red ovals) is then expressed, associates with and nicks the virion strand origin of replication (Step 2). As replication proceeds the old virion strand is progressively displaced (Steps 4 and 5). Following one fill cycle the displaced strand might be either released as a monomeric virus genome (Step 6a) or replication might continue for an additional cycle (Steps 6b and 7). Single stranded genome monomers and dimers yielded by Steps 6a and 7 can be converted to double stranded DNA by host DNA polymerases for additional rounds of replication. The genome length virion strands produced by Step 6a can also be encapsidated. Successive generations of virion strand DNA are colored blue, yellow and pink. After Jeske *et al*. [78].

In the circular ssDNA viruses, RCR can only commence following the conversion of ssDNA viral genomes into transcriptionally active covalently closed circular dsDNA molecules by host DNA polymerases (Step 1 in Figure 3). Once produced, dsDNA molecules probably associate with histone proteins and get packaged into mini-chromosomes suitable for gene transcription [69–71]. RCR begins when the expressed viral Rep protein site-specifically cleaves the virion strand at the virion strand origin (*v-ori*) [61,63,64,72–77] to produce an open circular “replicative form” DNA that becomes a template for continuous cyclical virion strand synthesis (Step 3 in Figure 3 [62,78]). As new virion strands are synthesized, the old strands are progressively displaced (Steps 4–7 in Figure 3) until, after one or more full circles, the ends of the old fully displaced strands are ligated to yield circular monomeric (Step 6a in Figure 3) or multimeric (Step 7 in Figure 3) ssDNA virion strands [72,74,77,78]. Re-circularized virion strands that are produced by RCR can be either encapsidated (but only if they are monomeric) or converted into covalently closed circular dsDNA by host polymerases for further rounds of replication [78].

Although it is presently unknown whether the anelloviruses also replicate via a rolling circle mechanism, circular dsDNAs have been detected in the livers and bone-marrow cells of people naturally infected with the anellovirus, Torque teno virus (TTV) [79,80]. Given that these DNAs are superficially similar to the replicative forms associated with RCR [58] and that the proposed origin of TTV replication contains highly conserved sequences that are vaguely similar to the origins of geminivirus, nanovirus and circovirus replication [81], it is plausible that anellovirus replication mechanisms may be similar to those of other circular ssDNA viruses.

**Figure 4.**
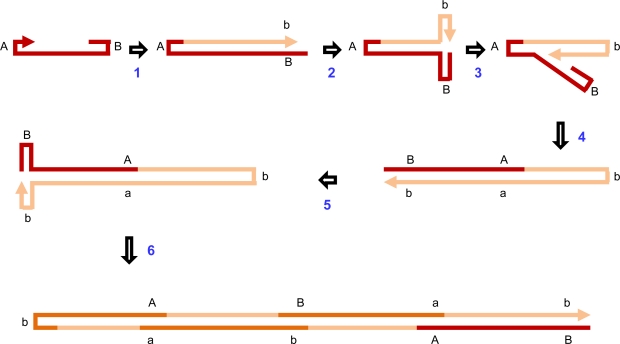
Rolling-hairpin replication in the parvovirus, Mouse minute virus. The linear unencapsidated parental parvovirus strand (in red) has a 3′ OH (indicated by the arrowhead) and palindromic ends that form terminal hairpin secondary structures (labeled A and B for the parental forms and a and b for the complements of the parental forms). The 3′ OH primes DNA synthesis in the direction of the arrow (Steps 1 and 2). When replication reaches the terminal B palindrome the replication complex switches strands and replication continues, producing a genome dimer (Steps 4 and 5). When it returns to the terminal B palindrome, the replication complex once again switches templates with replication continuing onwards to form a genomic tetramer (in dark orange and red) that is interspersed with redundant partial genome copies (in light orange). This double stranded form is then further processed to release genome length ssDNA molecules that are ready for packaging. From Cotmore and Tattersal [68].

Unlike in circular ssDNA viruses where complementary and virion strand synthesis occur in separate steps, the variants of RHR that occur in linear parvoviral genomes generally involve the replication of both viral strands during the same continuous process [68]. RHR is initially primed by the 3′ hydroxyl end of the 3′ terminal hairpin which initiates the production of a linear dsDNA molecule (Figure 4 the product of Step 1). Instead of simply ending at this point, the replication complex switches from the parental strand to the identical sequence on the newly synthesized strand and replication continues to produce a dsDNA molecule with paired hairpins at one end (Figure 4 the product of Step 2). This process then continues back and forth producing head to head and tail to tail genomic concatomers within which the palindromic genome ends are replicated half as frequently as the coding regions (Figure 4 the product of Step 4). New single stranded breaks introduced at the replication origins of these dsDNA molecules (in most cases by the parvoviral Rep homologue, NS1), result in the formation of new replication forks that, starting with the replication of the palindromic ends, displace ssDNA strands that are ready for packaging [62,68,82].

While the precise details of RCR and RHR mechanisms most likely vary quite substantially between the families (and even in many cases between the genera and species within individual families), from the perspective of understanding recombination mechanisms at least, the most important and completely conserved feature between these replication mechanisms is that they yield a variety of ds and ssDNA forms that often contain multiple genome copies.

### 2.2. Mechanisms of Homologous Recombination

The processes by which recombination occurs within ssDNA viruses are still quite poorly characterized but conceivably involve a number of different mechanisms. For example, interruption of replication by clashes between transcription and replication enzyme complexes or through single stranded breaks in the template strand of replicative form dsDNAs could cause premature detachment of replication complexes. If replication restarts following the reattachment of these complexes to template molecules other than those on which replication had initially started, the resulting fully replicated genomes will be recombinants generated by a mechanism known as copy-choice [83].

Another way in which recombination could potentially occur in ssDNA viruses is via host double stranded break repair pathways. Within cells infected with circular ssDNA viruses undergoing RCR many genomes will occur in the form of either covalently closed or open circular monomeric or multimeric dsDNA molecules [78] (Figure 2). Similarly, within cells infected with linear parvovirus genomes undergoing RHR numerous linear dsDNA viral genome concatomers will arise (Figure 3). When double stranded breaks occur within such molecules it is probable that they will induce generic host dsDNA break responses which will repair these molecules via homology dependent recombination mechanisms [78,84].

Starting from the broken ends of a dsDNA molecule these dsDNA break repair processes probably involve the 5′ to 3′ nuclease digestion (or resection) of the blunt ends to produce 3′ ssDNA overhangs. The 3′ ssDNA ends of broken molecules will then be “matched” up with homologous sequences on unbroken dsDNA molecules (a process known as strand-invasion) following which the 3′ ssDNA ends of the broken molecules will prime the synthesis of a new DNA strands on the unbroken templates [84].

When the template molecule is an unbroken covalently closed circular viral genome, the polymerase complexes producing the recombinationally replicated ssDNA strands can do multiple circuits around the template. These ssDNA strands can therefore potentially grow to three or more times the genome length and are presumably made double stranded by the same host mediated replication processes that convert ss viral DNA into dsDNA [78,85].

The so-called heterogeneous-length high molecular weight double stranded DNA (hDNA; [78]) molecules that this process yields can, in the case of geminiviruses and their associated DNA-beta satellites, comprise most of the viral DNA within infected cells. In these viruses it is therefore apparent that such “recombination dependent replication” (RDR) is a major mechanism of genome replication [78,85–88].

While it is unknown how common RDR is amongst ssDNA viruses, recombination patterns detectable within viruses isolated from nature display remarkable similarities across many of the rolling circle replicons [40,89] which suggest that groups such as the nanoviruses, circoviruses and even the prokaryote infecting microviruses might also replicate by RDR mechanisms. Similarly, just as has been noted in geminivirus infections, the high frequencies with which sub-full genome length DNAs occur in anellovirus infections (a symptom of frequent DNA breakage; [59]) suggests that these viruses too might replicate by a RDR mechanism.

When ssDNA viruses replicated by either RCR or RHR, produce hDNA molecules containing multiple genome copies and multiple replication origins (such as is expected in molecules that exceed twice the normal genome size), it is likely that “replicational release” of genome length virion strand molecules will occur from these just as it does from infectious tandemly repeated viral genomes that are frequently used to reconstitute circular ssDNA virus infections from cloned genome components [56,90,91]. These replicationally released genomes will contain one recombination breakpoint at the original site of strand breakage and, in the case of the circular ssDNA viruses, another at the origin of virion strand replication.

## 3. Component Reassortment

Whereas nanoviruses can have genomes consisting of up to eight circular ssDNAs each ∼1 Kb long [56,92–95], geminiviruses in the genus begomovirus can (but do not always) have genomes that consist of two components each ∼2.7 Kb long. Begomoviruses are also frequently associated with small ½ or ¼ genome length satellite molecules (Figure 1). Packaging of each genome component or satellite molecule within a different capsid and co-transmission to individual cells by insects of tens or perhaps even hundreds of virus particles means that the opportunities for genome component reassortment are probably rife. Such genome reassortments reconstituted from infectious clones are frequently infectious and, in the begomoviruses at least, can often produce distinctive (albeit usually quite mild) symptoms that, under the appropriate environmental conditions, might be selectively favored [96–102]. Accordingly, there are various known natural examples of components and satellites having been exchanged, lost or gained during the evolution of both the begomoviruses [100,103–107] and nanoviruses [47,108,109].

An important criterion that must be met for genome reassortants to be fully functional, however, is that their reassorted components and the genes they encode must interact efficiently with one another [96,110]. For example, the Rep and movement proteins expressed by a reassortant (Figure 1) must respectively trans-replicate and move all of its genome components.

Whereas in begomoviruses the DNA-B and DNA-beta components are trans-replicated by a Rep expressed from the DNA-A component, in nanoviruses it is a “master Rep” expressed from the DNA-R component that trans-replicates the other genome components. The replication origins of the various begomovirus and nanovirus genome components are structurally very similar and reside in a so-called common region that is usually quite highly conserved between the components of a given genome. The common regions of a genome’s various components contain repeated (or iterated) ∼5 nt long Rep specificity determinants (often called iterons) that are specific for the Rep expressed by that genome’s DNA-A (if it is a begomovirus) or DNA-R (if it is a nanovirus) [61,111–114]. In geminiviruses, nanoviruses and circoviruses a small ∼5 amino acid region near the N-terminus of Rep is responsible for recognition of its cognate specificity determinant sequences [12,115] implying that for a Rep to efficiently trans-replicate viral genome components these components must have common regions that contain compatible Rep specificity sequence elements [98,110,116].

Variation in the specificity with which different Reps interacts with the different genome constituents of multi-component viruses implies that there probably also exist variations in the rates with which different genome components are reassorted in nature. For example, geminivirus beta satellite molecules can be trans-replicated by Reps very distantly related to those of their cognate viruses [115,117–119] and it is likely that these molecules are therefore more prone to reassortment than DNA-B molecules that display a much greater degree of trans-replication specificity [103].

## 4. Inter-Component Recombination

It is probable that homologous recombination plays an important role in the production and adaptation of genome component reassortants. Given that DNA-A will rapidly and efficiently rescue a replication incompetent DNA-B component via homologous recombination [46,47,120,121], it is possible (if not probable) that the geminiviral DNA-A and the nanoviral DNA-R components maintain their replicational control over their dependent components by frequently updating the common regions of the dependent components by homologous recombination [46,47,120,121].

Similarly, when a component is introduced into a new genome, part of the capture process can involve replacement of the component’s common region with that of its new DNA-A / DNA-R component [46,47,104,113,122].

It is apparent, however, that simply replacing the common region of a dependent component with that of a new master component will not always produce an optimally functioning multi-component virus. For example, it has been shown in geminiviruses that shortly after a DNA-A captures a new DNA-B, the trans-replication process can be less efficient than that observed between co-evolved DNA-A/DNA-B component pairs [86]. Following component capture, there therefore likely exists a period of adaptive evolution that is required to optimize both the replicational and movement interactions between these components.

In both laboratory experiments and the field, recombination between the geminivirus DNA-A component and its associated satellites occasionally yields satellite molecules that have the DNA-A common region [62,105,123,124]. Although recombinant DNA-beta molecules carrying the DNA-A common region appear to be functionally similar to non-recombinant DNA-betas, their trans-replication will presumably be a lot more specific than that of “normal” DNA-betas.

It has been speculated that such recombinants may be a key stage during the formation of multi-component ssDNA virus genomes [103]. It is conceivable, for example, that the DNA-B component of geminiviruses may have arisen when non-homologous recombination between a DNA-A and a DNA-beta-like molecule yielded a trans-replication, movement and encapsidation proficient genome component prototype that contained the DNA-A intergenic region and coat protein gene and a DNA-beta-like symptom determinant gene [124]. Geminivirus DNA-B components encode a virion sense gene involved in nuclear trafficking of viral DNA (labeled NSP in Figure 1) and a complementary sense gene involved in transporting viral genomes from cell to cell (labeled MP in Figure 1; reviewed in [125]). The hypothesis that a recombination event between a DNA-A and a DNA-beta molecule could recreate a DNA-B-like component is credible because (1) both the single expressed gene on DNA-beta and the complementary sense gene of DNA-B encode symptom determinants [126–130]; (2) at least in some cases DNA-beta is able to substitute for the movement functions of DNA-B [131]; and (3) the DNA-B virion sense gene is both possibly a coat protein gene homologue and duplicates some of the coat protein’s nuclear trafficking functions [132].

## 5. Genome Rearrangement, Insertions and Deletions

Genomic rearrangements and deletions frequently arise during anellovirus [49,59,133], parvovirus [134–141] and geminivirus [21,123,134,141–145] infections.

Sub-full length genome molecules (hereafter referred to as sub-genomics) and genomes with sequence duplications that arise during natural parvoviral infections are possibly the result of DNA secondary structure induced template switching of DNA polymerases at 5–10 nt long direct repeats [137,140] during RHR. While the biological relevance of parvoviral sub-genomics and rearranged genomes are unknown, ultra-small sub-genomics retaining only the sequence elements required for trans-replication could potentially be harnessed for use as gene expression vectors [137].

In the anelloviruses, there is a tendency for sub-genomics to have breakpoints within either the 5′ half of ORF2 or at GC rich genomic sites [59]. Although most described anellovirus sub-genomics are unique and probably have no specific function, the frequent occurrence of small ∼560 nt long molecules containing the ORF I gene may indicate some special role for this category of sub-genomic during the virus life-cycle in species such as TTV [59]. However, since no strong associations have been noted between the presence of such sub-genomics and altered pathogenicity, the biological and/or evolutionary significance of these molecules remains uncertain.

By replicating at the expense of full-length genome components it is possible that sub-genomic DNAs that arise during geminivirus infections could reduce symptom severity and, in so doing, act as modulators of viral pathogenicity [146–149]. Although transgenic plants that are engineered to express geminivirus sub-genomic DNAs are usually more resistant to geminiviruses that are closely related to the sub-genomic transgenes than are comparable non-transgenics [150–152], they can also display increased sensitivity to infection [149].

In geminiviruses, sub-genomics can outnumber full length genomes by as many as ten to one [21] and while mostly involving straightforward viral sequence deletions and duplications they also frequently involve viral sequence inversions and insertions [21,123,147]. Regardless of their size and degree of rearrangement, however, almost all characterized geminivirus sub-genomic DNAs carry both the virion and complementary strand origins of replication [21,147] and can presumably be trans-replicated by their full-length counterparts.

While the significance of sub-genomic molecules in natural geminivirus infections remains unknown, it is likely that the same sequence deletion process that creates sub-genomics results in the rapid reversion to wild-type size of genomic DNA molecules with either insertions or deletions that make them bigger or smaller than full genome length [153–155]. The selection pressure on such size reversion is likely a strong size constraint on encapsidation [156]. This constraint is perhaps also illustrated by geminivirus associated alpha and beta satellites which contain A-rich genome regions ∼300 nts long that appear to be dedicated to modulating their size to maintain them within the range necessary to ensure their encapsidation within geminivirus virions [130]. To be persistently transmitted along with full length genomes, satellites and geminiviral sub-genomic molecules must apparently be approximately half full genome size to be suitable for encapsidation within half sized icosahedral (rather than usual geminate) particles [145,155–157].

Interestingly, small sequence deletions and rearrangements apparently occur at very high frequencies near the *v-ori* of circovirus genomes and can very rapidly optimize both the rearrangement of disrupted iterated Rep binding site sequences [158] and the lengths of artificially shortened inverted repeat sequences within the *v-ori* hairpin structure [63]. While not as obvious as the large sequence deletions, insertions and rearrangements seen in parvoviruses, geminiviruses and anelloviruses, the more subtle versions of these sequence modifications seen in circoviruses may also play a crucial but unrecognized role in preserving the replication origins of many other RCR replicons.

## 6. Recombination between Viral and Host Genomes

The recombinational transfer of genetic material between ssDNA viruses and their hosts is known to occur in both directions. Geminivirus sub-genomic and satellite molecules occasionally contain small fragments of non-viral DNA that is presumably host derived [21,105]. The fact that adaptive recombinational transfers of DNA from host genomes can occur has been definitively demonstrated in experiments examining the transfer of coat protein transgenes from host genomes into geminivirus genomes lacking a functional coat protein [159].

Non-homologous recombination between host and viral DNA occasionally results in the integration of ssDNA virus genome fragments into host chromosomes [55,160–162]. Some variants of the human infecting parvovirus species, adeno-associated virus (AAV), frequently integrate at a specific site on chromosome 19 [163]—a feature that makes these viruses particularly promising as potential gene therapy vectors. Scans of eukaryote full genome sequences have revealed that there have likely been numerous instances during the past when other ssDNA virus genomes have also become stably integrated within the genomes of various animal, plant and prokaryote species (see orange branches in Figure 2; [55,160]). For example, it is probable that two integrations of geminivirus genomic DNA probably occurred between 0.2 and 5 million years ago (MYA) within the ancestral germ line(s) of certain *Nicotiana* species: One integration in the ancestral germ line of *Nicotiana tabacum*, *N. tomentosa*, *N. tomentosiformis* and *N. kawasamii* and another in that of *N. tabacum* and *N. tomentosiformis* [164–169].

Integrated viral genomes are interesting because they provide a view of what ssDNA viral genomes may have looked like millions of years ago and can enable us to determine the timing of important events during the evolution of these viruses. For example, despite extremely rapid short term evolution rates [15,24] that imply that these families could plausibly have arisen less than 1 MYA, strong evidence for the ancient integration of some circoviruses and parvoviruses within the ancestral lineages of multiple extant species strongly suggests that these viral families respectively arose more than 55 and 98 MYA [160,162]. Similarly, the geminivirus sequences integrated into the *Nicotiana* genome have been used as evidence to indicate that distinct geminivirus lineages present in the New and Old Worlds are less likely to have diverged following the breakup of Gondwanaland 100 MYA [170] than to have diverged following the climate-change induced closure of a temperate and quite expansive land-bridge between Asia and North America around 30 MYA [169].

It is also very probable that as more eukaryote genomes are sequenced additional integrated ssDNA virus genome fossils will be uncovered. For example, during our construction of the Rep gene phylogeny presented in Figure 2, we discovered two previously unreported instances of geminivirus-like sequences within the genomes of the dark cotton wood tree and the common apple.

## 7. Ecological and Epidemiological Influences on Patterns of Recombination between ssDNA Virus Populations

The rates of recombination and genome reassortment that occur between different viral species or strains will obviously be strongly influenced by the frequency with which viruses in these different lineages co-replicate within the nuclei of shared host species. Therefore degrees of overlap between (1) geographical ranges, (2) epidemiological cycles, (3) host ranges, and (4) tissue tropisms are all expected to be major determinants of how frequently the individuals within different virus populations recombine with one another. Most well characterized terrestrial ssDNA virus species with non-human hosts seem to have well defined geographical ranges with populations within these species displaying high degrees of spatial structure [89,171–176]. What this means is that even within particular ssDNA virus species where all individuals have perfectly overlapping host ranges, tissue tropisms and epidemiological cycles, there will probably not be equal opportunities for all individuals to recombine with one another.

Epidemiological factors that will determine how frequently viruses that could feasibly recombine actually do recombine will be the numbers of individuals infected with the viruses, the viral titers that are attained within these infected individuals and the durations of their infection cycles. Whereas numbers of infected individuals will determine the frequency with which genetically distinct viruses co-occur within mixed infections, viral titers and infection durations within co-infected individuals will determine the frequency with which the viruses co-occur within the same infected nuclei. For example, it is probable that the large numbers of recombinants observed in many anellovirus species are due to these species having extraordinarily high incidences (often >75%) within their respective host populations [49,177,178].

Finally, the host range sizes of ssDNA viruses will determine how many opportunities they have to recombine with related species in the environment. A good example of how variation in host ranges can influence opportunities for recombination can be found amongst the geminiviruses of South America. On this continent, an enormous number of geminivirus species have emerged as crop pathogens within the last two decades. This surge in the number of reported geminivirus associated plant diseases has been widely attributed to the introduction onto the continent in the early 1990s of a highly invasive transmission vector strain (or biotype) that feeds on a far wider range of species than indigenous strains [179–181]. By breaking the transmission barriers that had previously existed between many potential geminivirus host species, the new vector has increased the effective host ranges of enormous numbers of indigenous South American geminiviruses [179]. Therefore, whereas only a single geminivirus species had been reported in Brazil as a tomato pathogen prior to 1990, today the list of recognized Brazilian tomato pathogens includes fifteen geminivirus species [182]. While there is no definitive evidence that broadened host ranges have caused increased rates of recombination amongst the South American geminiviruses, it is nevertheless interesting that most of these newly discovered tomato-infecting species are obvious recombinants [182–187].

## 8. Mechanistic Influences on Homologous Recombination Patterns

When genetically distinct ssDNA virus genomes co-replicate within the same nucleus there are a number of different mechanistic factors that could determine the patterns of recombination that might occur. Recombination breakpoints that are detectable in ssDNA virus genomes sampled from nature are generally not randomly distributed and have been known to either cluster within discrete recombination hot-spots or to occur much less frequently within recombination cold-spots [36,40,55,188–190]. These uneven breakpoint distributions are in many cases caused by underlying differences in the rates at which recombination occurs in different parts of ssDNA virus genomes. Mechanistic factors that might influence site-to-site variations in basal recombination rates across these genomes include the locations of replication origins, degrees of sequence similarity between recombining genomes, genomic ssDNA secondary structures, the orientations of genes in relation to directions of rolling circle replication, and differential degrees of dsDNA exposure within histone packaged viral mini-chromosomes.

### 8.1. Replication Origins

Full genome sequence analyses of field isolated circular ssDNA viruses that replicate via RCR have indicated that the *v-oris* of almost all of these (excluding the nanoviruses) are recombination hot-spots [40]. These *v-ori*s are defined by a 10–30 nt long inverted repeat sequence capable of forming a hairpin structure that contains within its loop a highly conserved nonanucleotide sequence that defines the actual *v-ori* [62,64,191].

Recombination experiments in geminiviruses [192] and circoviruses [193], have shown that this genome site is a mechanistically predisposed recombination hot-spot because of the replicational release of viral genomes from genomic concatomers (Figure 3). When these concatomers either arise following a copy-choice mediated polymerase strand switch, or break and are rescued by host double stranded break repair pathways (*i.e.*, by recombination dependent replication [78]), recombinants that are replicationally released will have one breakpoint at the site of the strand-switch/breakage and another at the *v-ori* (see Section 2.2 above).

### 8.2. Sequence Similarity

The efficiency with which homologous recombination can be used to replicationally repair broken ss and dsDNA molecules is strongly dependent on the degrees of similarity between broken sites and those of the unbroken molecules used as templates during recombinational repair. Obviously when the broken molecules and their homologous templates are 100% identical this consideration is irrelevant. However, in a mixed infection when the template and broken molecule are not identical, it is expected that recombination will tend to occur most efficiently if the break occurs within a genome region where the broken and template molecules are very similar to one another. This is in fact what is observed during *in vitro* recombination experiments [169,194]. Curiously, in geminiviruses it has been found both that recombination breakpoints can occur (albeit at quite a low frequency) between two nucleotides that are non-identical in both parents and that recombination tends to occur more frequently at genome sites where the two parental genomes share between 5 and 14 identical nucleotides than at sites where they share longer runs of identical sequence [169].

### 8.3. ssDNA Secondary Structure

In many viruses with ssRNA genomes, secondary RNA structures colocalize with recombination hot-spots [195–200]. This is probably because they cause replication complexes to momentarily stall and, in so doing, increase the probability of strand transfers that can cause the copy-choice type of recombination. The *v-ori* of most circular ssDNA viruses is both a recombination hot-spot and forms a stable hairpin structure and it is conceivable that these two characteristics are not unrelated. In circoviruses, for example, a very credible “melting pot” hypothesis has been proposed to explain how secondary structure in this region facilitates local copy-choice recombination within the stem of the hairpin so as to maintain its palindromic sequence [63].

Computational predictions indicate that additional uncharacterized ssDNA structures probably exist within many ssDNA virus genomes [194,201] and the possibility exists that these too may facilitate recombination [59,194]. For example, whereas in the anellovirus, TTV, there is a tendency for recombination to occur within GC rich regions that have the potential to form secondary structures [59], in some geminiviruses it has been noted that recombination breakpoints falling outside the *v-ori* hairpin tend to occur at sites where one parental sequence has a predicted secondary structure but the other does not [194].

It should also be pointed out here that, as we explain later, genomic secondary structures may have another quite different influence on where recombination breakpoints occur within geminivirus genomes. Specifically, it is apparent that whereas the overall genomic secondary structures of recombinant genomes can vary quite substantially from those of their parents, there is evidence of strong selection pressures in geminiviruses for recombinants to maintain parent-like secondary structures [189].

### 8.4. Transcription-Replication Clashes

ssDNA viruses such as the geminiviruses and circoviruses have genes that are expressed from both the virion and complementary sense strands. It is perhaps significant that the complementary sense genes of these viruses are expressed in the opposite direction to RCR because whenever DNA replication proceeds in the opposite direction to gene transcription the opportunity exists for replication complex-transcription complex clashes [202]. Evidence of such clashes during geminivirus and circovirus replication is that the members of these families tend to have more detectable recombination events and measurably higher estimated population-scaled recombination rates in their complementary sense genes than they do in their virion sense genes [78,83,107,203]. The imbalance between recombination rates in the virion and complementary sense genes in these viruses is particularly apparent when considering only recombination occurring between very closely related sequences [40,83]. This suggests that, in these viruses, strongly homology dependent copy-choice recombination may be a particularly important mechanism of replication re-initiation following interruption due to transcription-replication complex clashes.

### 8.5. Differential Degrees of ssDNA Exposure within Mini-Chromosomes

Another factor that influences site to site variations in basal recombination rates across ssDNA virus genomes is the association of transcriptionally active viral covalently closed circular DNA forms with host histones and their packaging into mini-chromosomes. Although mini-chromosome formation amongst the ssDNA viruses has only been definitively shown for parvoviruses [204] and geminiviruses [69–71], it is probable that all the other nuclear localized eukaryote infecting ssDNA viruses also form such structures during the transcriptionally active portions of their life-cycles.

Recombination breakpoint hot-spots detectable within the genomes of geminiviruses in the genus begomovirus co-localize very closely with genome sites that are exposed within mini-chromosomes to host transcription and replication factors [71,78,107]. These sites are also apparently the most common sites of dsDNA breakage during begomovirus infections [78] and it is therefore likely that at least part of the reason that these regions are recombination hot-spots is that they are hyper-sensitive to either physical breakage or host nuclease attack.

## 9. The Adaptive Value of Recombination in ssDNA Viruses

As with most other organisms on Earth it is likely that recombination between pairs of nearly identical ssDNA viruses is both a key mechanism in rescuing broken genomes [78] and an effective defense against the otherwise unavoidable accumulation of deleterious mutations [205–207]. As with many other recombining viruses, recombination between more distantly related genomes could also potentially provide ssDNA viruses with far more adaptive options than are attainable through mutation alone.

It is expected that if such adaptive recombination occurs, the recombinants that it yields should increase in prevalence to the point that they become detectable as circulating recombinant forms (CRFs; *i.e.*, when multiple examples of the same recombinant forms have been sampled from the environment they are classified as CRFs). Although the detection of CRFs in many circovirus [40,43,208–210] parvovirus [40,54], geminivirus [36,89,107,171,211] and anellovirus [40] species strongly suggests that many recombination events between viruses in these families might be adaptive, there is very little available direct evidence to support this view.

There are nevertheless various instances where recombination amongst geminiviruses has been circumstantially implicated in the alteration of host ranges and the pathogenicity. Among others these include the emergence of Maize streak virus as an important agricultural pathogen in the mid-1800s [89,212], the dramatic increases in the severity of cassava mosaic disease seen in central-east Africa during the 1990s [33,35,100,213,214]; the emergence of diverse economically relevant begomoviruses in South America [187] and the Indian subcontinent [34,215] during the 1990s and the invasive spread of tomato yellow leaf curl disease causing viruses across the Western Mediterranean during the past three decades [216–218].

Laboratory constructed geminivirus and parvovirus recombinants have revealed that recombination can potentially alter transmission vector specificities [219,220], degrees of pathogenicity [192,221,222], host ranges [216,223], degrees of neutralizing antibody resistance [224–226] and tissue tropisms [221,224,227–230].

The adaptive potential of recombination has in fact been definitively proven in the *in vitro* evolution under artificial selection of parvovirus-based gene delivery vectors. For example, the parvovirus, AAV, could be particularly useful for the delivery of genes to treat hereditary heart muscle degenerative diseases. Since no known natural variants specifically infect only heart muscle cells, these variants have been artificially “bred” using *in vitro* selection of randomly generated coat protein gene recombinants within muscle cells [229].

It has also been experimentally demonstrated in geminiviruses that recombination can be highly adaptive. In mixed infections of mutant viruses that collectively have the genetic material to produce fully viable genomes, within a few weeks “repaired” recombinants emerge [231]. Similarly, when viable but severely defective laboratory constructed recombinants occur together within mixed infections, genomes closely resembling those of wild-type viruses emerge very rapidly in multiple repeated experiments [21]. This rapid deterministic convergence on what may be a near optimal “recombinant solution” to the problem of optimizing virus fitness given a set of differentially adaptive parental polymorphisms, suggests, firstly, that during geminivirus infections a very wide variety of recombinants are generated and, secondly, that selection very efficiently sorts these for genomes with increased fitness.

## 10. Selective Constraints on the Adaptive Value of Recombination

Besides demonstrating the adaptive potential of recombination, experiments evaluating the viability of laboratory constructed genome chimeras have also revealed that most natural recombination events that occur between ssDNA viruses are either neutral [232] or at least slightly maladaptive, yielding progeny genomes that are on average less viable than their parents [111,142,192,222,233,234]. Therefore, besides the mechanistic factors that cause variations in basal recombination rates across ssDNA virus genomes, recombination breakpoint distribution patterns are probably also at least partially attributable to natural selection disfavoring the survival of many (if not the vast majority) of the recombinants that arise during mixed infections. It is fairly obvious that unless a newly generated recombinant can productively compete with its parents in terms of replication rate, systemic movement and transmission, it will generally not survive for long enough to become an independent viral entity.

A good example of the competitive hurdles that recombinants must overcome on their path to emergence can be found in the Tomato infecting geminiviruses of Sicily and Spain. Two species, tomato yellow leaf curl virus (TYLCV) and Tomato yellow leaf curl Sardinia virus (TYLCSV) frequently coexist within mixed tomato infections in these countries and yield a variety of recombinant forms. Although at least some of these recombinants have proven viability [216,233,235] and are very similar to other recombinants found in many different field [216,217,233] and reconstituted laboratory [194,236,237] TYLCV and TYLCSV mixed infections, it is apparent that they may require very specific ecological conditions to survive on their own in nature [233]. Whereas in Spain some of these recombinants occur on their own, in Sicily they are only ever found within mixed tomato infections with one or both of their parental viruses. Potential differences in vector transmissibility aside, this is probably at least partially attributable to the fact that many of the recombinants are less infectious in tomato and replicate to lower titers than either TYLCV and TYLCSV in this host [233]. In Spain, where some TYLCV-TYLCSV recombinants are found on their own [217], they are either fitter than, or as well adapted as, their parents when it comes to infecting alternative hosts such as black nightshade (*Solanum nigrum*, an uncultivated species common in the tomato growing regions of Spain; [235]) or common bean (*Phaseolus vulgaris*, a crop which are frequently grown in rotation with tomatoes in Spain but not in Sicily; [216,217,233]).

Furthermore, an interesting feature of the most prevalent and widely distributed of the TYLCVTYLCSV recombinants that have emerged in Spain in recent years is that it has apparently experienced elevated rates of non-synonymous nucleotide substitution relative to its parental virus lineages [217]. As has been indicated experimentally with laboratory constructed recombinants of prokaryote infecting ssDNA microviruses [238], this observation supports the hypothesis that following natural recombination events there likely exists a period during which the different portions of recombinant genomes must adapt to one another. An important factor determining the adaptive value of recombination may therefore also be the accessibility of compensatory mutations that will reverse its fitness costs [238].

It is probable that the fitness costs associated with recombination are due at least in part to recombination frequently disrupting co-evolved intra-genome interaction networks [238]. These “favorable epistatic interactions” might be nucleotide-nucleotide interactions within nucleic acid secondary structures [189], encoded amino acid interactions within folded protein structures [224,239], or sequence specific protein-protein or protein-DNA interactions that form the basis of longer-range intra-genome interactions [189,240].

### 10.1. Disruption of Long-Range Intra-Genome Interactions

It has been noted in geminivirus recombinants that when parental viruses each have different Rep recognition sequences near the *v-ori*, the Rep recognition sequences and the N-terminal region of Rep (which encodes the recognition sequence binding site) tend to always be inherited from the same parent. This pattern is consistent with the hypothesis that recombinants with incompatible Rep/Rep binding site sequence pairs will in most cases be replicationally defective [215]. Accordingly, laboratory constructed recombinants are generally either unviable or severely attenuated when Rep encoding genes are transferred into genomes that have incompatible Rep specificity determinants [241–243].

By comparing the viability of pairs of laboratory constructed recombinant viruses with reciprocal gene exchanges to the viability of their non-recombinant parents, it is clear that there are likely numerous such interactions throughout ssDNA virus genomes that must be maintained to ensure the fitness of recombinant genomes [142,221,222,234]. Specifically, the average viability of such reciprocal recombinant pairs is almost invariably less than the average of their parents. If no long-range interactions were disrupted during the construction of such recombinants then one would expect their average viability to be approximately equal to that of their parents [240].

The degree of disruption that recombination causes in long-range interactions depends on both the genome regions that are transferred and the degree to which the transferred sequences resemble those that they replace [240]. As a general rule it is expected that genome regions that do not interact in a sequence-specific manner with other viral genome regions tend to be quite modular. What this means is that these regions will tend to function better within the context of foreign genomes than genome regions that have extensive interactions with either viral sequences or the proteins they express. Accordingly, recombinational transfers of less interactive genome regions are expected to incur lower fitness costs than transfers of more interactive regions.

Regardless of how interactive a transferred genome region is, if the portion of its sequence that is involved in these interactions is identical in both parental viruses such that no interactions are disrupted, then it is likely to continue functioning well following its transfer [238]. Therefore, one expects that while productive transfers of less interactive genome regions could occur between distantly related viruses, productive transfers of more interactive genome regions will tend to occur only between more closely related viruses with high degrees of genetic compatibility in these regions [232,240].

The constraints that mandatory maintenance of optimal long-range sequence specific interactions place on the viability of recombinants are so severe that patterns of recombination that arise during recombination can actually be used to trace these interactions. Specifically, within the recombinant genomes that arise during a mixed infection of the tomato infecting begomoviruses TYLCV and Tomato leaf curl Comoros virus (TLCCV) there is a significant tendency for genome regions that interact with one another to be inherited from the same parent [189]. This tendency is surprising in that the infectivity and replication efficiency of randomly generated TLCCV and TYLCV recombinants is not obviously different from that of the wild-type viruses [232]. Nevertheless, the subtle fitness difference between the recombinant viruses are profound enough that given 50 unique recombinants arising during independent mixed infections of TYLCV and TLCCV it has proven possible to use pair-wise nucleotide association mapping to retrace every long-range intra-genome interaction that is known to occur within begomovirus genomes.

### 10.2. Disruption of Protein Folding and Oligomerization

Another class of interactions that could potentially be disrupted by recombination are the amino acid interactions that are required for proper protein folding and/or oligomerization. Whereas many amino acids within a protein must specifically interact with one another to ensure proper folding [244], protein oligomerization can also require specific interactions between different groups of amino acids encoded by a single gene. It is possible that at least part of the reason that almost all analyzed ssDNA virus groups display more recombination breakpoints within intergenic regions than within genes [40,190] is that recombination breakpoints within coding regions tend to be more deleterious than those in non-coding regions. Consistent with this view is that the recombination breakpoints that do occur within the genes of ssDNA viruses tend to cluster on the edges of the genes where they are expected to have the lowest impact on intra-protein amino acid interactions [40]. Also, genes encoding the highly oligomeric coat proteins of ssDNA viruses [40] generally accumulate fewer recombination breakpoints than other viral genes indicating that the likelihood of recombination within a gene disrupting favorable intra-protein amino acid interactions increases if the gene encodes a protein that forms complex oligomers (for example, those forming viral capsids).

In geminiviruses and parvoviruses the impact of recombination induced disruption of protein folding has in fact been directly detectable within the replication associated protein and coat protein genes of recombinants arising both in the field [239] and during controlled evolution experiments [189,224]. Analyses of chimeric parvovirus coat proteins have clearly demonstrated that while recombination can seriously damage amino acid interactions necessary for proper capsid assembly [245], preservation of these interactions is the most important factor determining breakpoint patterns found within viable coat protein recombinants [224]. Similarly, the geminiviruses atomic resolution 3D structure models of Rep and CP have enabled the estimation of degrees of folding disruption within the chimeric proteins expressed by both real and simulated recombinant viruses. The fact that real recombinants tend to express proteins with significantly lower degrees of estimated protein folding disruption than randomly generated simulated recombinants strongly supports the notion that chimeric proteins are frequently misfolded and that natural selection strongly disfavors the survival of recombinant viruses that express such proteins.

### 10.3. Disruption of Genomic Secondary Structure

In much the same way as recombination between divergent ssDNA viruses can potentially disrupt protein folding when breakpoints occur within protein coding regions, it could potentially disrupt the folding of ssDNA genomes into biologically important secondary structures. Although the importance of genomic secondary structures at the replication origins of many ssDNA viruses is well established [246–249], it is possible that there exist additional biologically relevant secondary structures throughout many, if not all, ssDNA virus genomes [189,201,250]. In geminivirus recombination experiments, the inferred secondary structures of recombinant genomes that emerge during mixed infections are generally far less disrupted relative to parental genomes than are those of computationally generated recombinants [189]. This strongly implies that, just as natural selection disfavors the survival of genomes that express misfolded chimeric proteins, it also disfavors the survival of recombinant genomes with misfolded secondary structures.

## 11. Conclusions

Given the prominence of recombination during the evolution of ssDNA viruses it is reasonable to speculate that their basic genome organizations have most likely evolved to both maximize the adaptive value of recombination and minimize its potentially deleterious effects. For example, the central role of recombination throughout the evolutionary histories of the ssDNA viruses that replicate via RCR is probably reflected in the genomic positioning of their Rep genes and the genome sites that interact with Rep. Generally, replication specificity determinants that interact with Rep and the nucleotide sites encoding the Rep residues that interact with these specificity determinants are within 100 nucleotides of one another and are frequently bounded by recombination hot-spots. This genomic arrangement ensures that, following the transfer of this “replication specificity module” into a foreign genomic background, there is a high probability that it will continue to function properly. As has been indicated in various studies [189,224], simple statistical tests for the presence of such modules within recombinant ssDNA virus genomes could provide us with a straightforward means of detecting many of the other sequence specific intra-genome interactions that underpin the biology of these viruses.

While the examination of ssDNA virus recombination patterns at the genome scale could provide valuable insights into the genetic architectures of these viruses, use of virus sequence data to estimate recombination rates and patterns of sequence exchange at the population scale could be a powerful means of comparatively studying the epidemiological characteristics of ssDNA virus populations. Specifically, differences between intra-population recombination rates of otherwise very similar virus populations would indicate differences in the rates at which viruses in the populations co-infect individual nuclei. Coupled with incidence and viral load data, recombination rate estimates could be used to differentiate between infection incidences, virus titers within infected individuals, and differences in infection durations as causes for differences in the recombination rates. Such data could prove crucial for modeling epidemiological influences on the recombination patterns seen in ssDNA viruses [251].

While certainly informative, it can also be problematic to directly compare such “population-scaled” recombination rate estimates between different species. It would therefore be very valuable if efforts were made to directly estimate for different ssDNA viruses in the absence of any selection both basal per replication cycle recombination rates and basal genomic site-to-site variations in recombination frequencies. With such data in hand it will be possible to control for mechanistic differences in recombination between species so as to increase the power with which recombination patterns can be used to either identify the intra-genomic sequence interactions that are most strongly preserved by natural selection or infer ecological interactions between species.

Since recombining viruses obviously have somewhat overlapping geographical distributions, host ranges, and tissue tropisms, patterns of sequence exchange amongst viruses sampled from nature could also be used to map the ecological interactions between virus species. Whereas the web of genetic exchanges amongst viruses could reveal the virus lineages that are the most promiscuous recombiners (and which are therefore likely to be major contributors to future recombinants), within the context of host species preferences and geographical ranges, such maps could also reveal key environments (such as equatorial regions or temperate grasslands) and host species (such as widely dispersed wild grasses or domesticated farm animals) where recombination occurs most frequently. From a purely disease control perspective such information could be extremely valuable when it comes to making policy decisions aimed at reducing the probabilities of dangerous recombinants emerging. Such undesirable recombinants could include those that are more virulent, break inbred/genetically engineered resistance genes, are drug resistant and evade vaccine induced immune responses.

As is the case with many RNA viruses, the evolution, dispersal, and population growth/decline rates of ssDNA viruses all occur within similar time-frames such that histories of movements and population size variations of many ssDNA virus species should be detectable using their genomic nucleotide sequences. Besides potential applications of temporally scaled phylogenetic analyses to the dating of important evolutionary events such as host range or vector preference switches, recently developed phylogenetics based approaches could be used to pinpoint the geographical locations where these events occurred [218,252,253], determine whether they were associated with altered adaptive evolution rates [176,217], and, based on the phenotypic information gathered from sampled contemporary viruses, indicate what the likely biological effects of these evolutionary events were [254,255]. Coupling of such analyses with advances in both the computational inference of ancestral recombinant virus genome sequences [256] and cheap DNA synthesis means that the opportunity now exists for us to literally recreate infectious ancestral recombinant virus genomes [257] and use these to directly determine things like their neutralization susceptibilities, host ranges, tissue tropisms and vector specificities. Put simply, from the perspective of studying recombination, these new analytical techniques have finally provided us with the tools to definitively determine the adaptive value in ssDNA viruses of this important evolutionary process.
